# Chian Turpentine

**Published:** 1880-10

**Authors:** 


*Chian Turpentine*, seems from a report in the September issue of the *Virginia Medical Monthly*, to be acting charmingly in a case of carcinoma uteri, in Richmond, and it is sincerely hoped, that the encouraging effects that have been thus far seen, may be continued until complete eradication of the disease is produced. We await further information with deep interest in the final result.

